# Key LncRNAs Associated With Oxidative Stress Were Identified by GEO Database Data and Whole Blood Analysis of Intervertebral Disc Degeneration Patients

**DOI:** 10.3389/fgene.2022.929843

**Published:** 2022-07-22

**Authors:** Xueliang Jiang, Junfei Wu, Chunhui Guo, Wenhui Song

**Affiliations:** Department of Orthopaedic Spinal Surgery, The Second Hospital of Shanxi Medical University, Taiyuan, China

**Keywords:** intervertebral disc degeneration (IDD), long noncoding RNA (lncRNA), oxidative stress (OS), competing endogenous RNA (CeRNA), immune landscape

## Abstract

**Background:** Intervertebral disc degeneration (IDD) is a major cause of low back pain, but the onset and progression of IDD are unknown. Long non-coding RNA (lncRNA) has been validated to play a critical role in IDD, while an increasing number of studies have linked oxidative stress (OS) to the initiation and progression of IDD. We aim to investigate key lncRNAs in IDD through a comprehensive network of competing endogenous RNA (ceRNA) and to identify possible underlying mechanisms.

**Methods:** We downloaded IDD-related gene expression data from the Gene Expression Omnibus (GEO) database and obtained differentially expressed-lncRNAs (DE-lncRNA), -microRNAs (DE-miRNA), and -messenger RNAs (DE-mRNA) by bioinformatics analysis. The OS-related lncRNA-miRNA-mRNA ceRNA interaction axis was constructed and key lncRNAs were identified based on ceRNA theory. We performed gene ontology (GO) and Kyoto Encyclopedia of Genes and Genomes (KEGG) pathway analyses on mRNAs regulated by lncRNAs in the ceRNA network. Single sample gene set enrichment analysis (ssGSEA) was used to reveal the immune landscape. Expression of key lncRNAs in IDD was assessed by quantitative reverse transcription-polymerase chain reaction (qRT-PCR).

**Results:** In this study, 111 DE-mRNAs, 20 DE-lncRNAs, and 502 DE-miRNAs were identified between IDD patients and controls, and 16 OS-related DE-lncRNAs were also identified. The resulting lncRNA-miRNA-mRNA network consisted of eight OS-related DE-lncRNA nodes, 24 DE-miRNA nodes, 70 DE-mRNA nodes, and 183 edges. Functional enrichment analysis suggested that the ceRNA network may be involved in regulating biological processes related to cytokine secretion, lipid, and angiogenesis. We also identified four key lncRNAs, namely lncRNA GNAS-AS1, lncRNA MIR100HG, lncRNA LINC01359, and lncRNA LUCAT1, which were also found to be significantly associated with immune cells.

**Conclusion:** These results provide novel insights into the potential applications of OS-related lncRNAs in patients with IDD.

## Introduction

Intervertebral Disc degeneration (IDD) is a common pathologic change in the intervertebral disc. It is directly related to lower back pain, which causes great pain to patients and great financial pressure on society (Risbud and Shapiro, 2014). In China, low back pain is gradually becoming an epidemic. Statistics show that the number of patients with low back pain has exceeded 200 million, accounting for about 16% of the country’s population.

The underlying pathogenesis of disc degeneration is not fully understood. We know that it is not only related to external factors such as obesity and fatigue, but also to genetic factors. These factors can lead to changes in cell morphology, apoptosis, senescence, inflammation and oxidative stress (Roh et al., 2021). However, these currently known mechanisms do not provide good results for the treatment of IDD, so further research is needed to develop more effective prevention and treatment strategies.

Noncoding RNAs include long non-coding RNAs (lncRNAs), mirco RNAs (miRNAs) and circular RNAs (circRNAs). LncRNAs, with a length of over 200 nucleotides, can’t encode proteins, but it plays a regulatory role in biological processes such as proliferation, invasion and apoptosis and deregulates inflammation and infection (Hausser and Zavolan, 2014). LncRNA may act as competing endogenous RNAs (ceRNAs) to regulate the expression of target mRNAs through competitively binding to mRNAs. It’s pivotal to illustrate interactions of lncRNAs, miRNAs and mRNAs for treatment of IDD (Salmena et al., 2011). With the development of the society, more and more studies have been conducted to verify the hypothesis. A recent study shows that (Ma et al., 2021) MIR155 host gene (MIR155HG), a long non-coding RNA, promotes cell pyroptosis. In human degenerative NP tissue samples, MIR155HG expression was significantly increased and positively correlated with the Pfirrmann score. Excessive expression of MIR155HG reduces the miR-223-3p level, upregulates NLRP3, and promotes cell pyroptosis in human degenerative NP cells. The interaction of MIR155HG, miR-223-3p, and NLRP3 is like the mode of ceRNA (Yang et al., 2022). We know that lncRNA HLA complex group 18 (HCG18) also has its own ceRNA network to induce the development of IDD (Xi et al., 2017). The regulatory function of ceRNA provides a new perspective for elucidating the gene expression regulatory network constructed by transcriptome, and provides more dimensions for analyzing the molecular mechanisms of important biological processes. Intervertebral disc degeneration is closely related to intervertebral disc senescence, and oxidative stress is the main factor causing cell senescence. Nucleus pulposus cells are one of the main sources of reactive oxygen species (ROS), and ROS levels in intervertebral discs increase with the progression of intervertebral disc degeneration (Brandl et al., 2011). Hydrogen peroxide can activate the senescence signal transduction pathway and induce cell cycle stagnation in G0/G1 phase of nucleus pulposus cells. These studies indicate that oxidative stress is an important inducement of intervertebral disc degeneration (Yudoh et al., 2005; Wang et al., 2021a).

We aimed to identify DE-lncRNA, DE-miRNA, and DE-mRNA between an IDD group and a control group by bioinformatics analysis, then a ceRNA network was made, and through GO and KEGG pathway analyses, the underlying function was revealed. The present study researched the ceRNA network and how to regulate biological processes associated with cytokine secretion, lipid, and angiogenesis and found four key lncRNAs, lncRNA GNAS-AS1, lncRNA MIR100HG, lncRNA LINC01359, and lncRNA LUCAT1, which were also found to be significantly associated with immune cells. QRT-PCR was used to certify it. This study may provide new therapeutic strategies and novel insights into the potential applications of OS-related lncRNAs for IDD.

## Materials and Methods

### Data Source

IDD-related gene expression profile data were obtained from the GEO database. mRNA and lncRNA expression profiles were obtained from the GES124272 dataset (platform:GPL21185; https://www.ncbi.nlm.nih.gov/geo/query/acc.cgi?acc=GSE124272), which contains eight patients with MRI-confirmed IDD and eight volunteers (no clinical evidence of low back pain or sciatica) of fasting whole blood (collected from the left median cubital vein of each participant) samples (Wang et al., 2019; Wang et al., 2021b). The miRNA microarray of three IDD patients [nucleus pulposus (NP) tissues] and three controls (normal NP tissues from fresh traumatic lumbar fracture patients) was obtained from the GSE116726 dataset (platform: GPL20712; https://www.ncbi.nlm.nih.gov/geo/query/acc.cgi?acc=GSE116726) (Ji et al., 2018).

GeneCards online tool (https://www.genecards.org/) was utilized to obtain oxidative stress-related genes (OSRG). Briefly, using the keyword “oxidative stress” and setting the “Category” to “Protein Coding”, filtered genes based on Relevance score ≥ 7, and finally, 2,129 OSRGs were obtained (Supplementary Table S1).

### Differential Expression Analysis

The differential expression analysis was performed by R package limma. The significance threshold was set at |log_2_ fold change (FC)| ≥ 1 and *p* < 0.01. The basis of DE-mRNAs and DE-lncRNAs analysis was from the GSE124272 dataset and DE-miRNAs were identified in the GSE116726 dataset. Volcano plots were drawn using the R package ggplot2 to demonstrate the expression distribution of DE-mRNAs, -miRNAs, and -lncRNAs.

### Identification of Oxidative Stress-Related Differentially Expressed-lncRNAs

After matching the expression profiles of 2,129 OSRGs in the GSE124272 dataset, the relationships between all lncRNAs in the GSE124272 dataset and OSRGs were calculated based on the expression values. OS-related lncRNAs were identified based on Spearman correlation coefficients with absolute values >0.8 and *p* < 0.01. Subsequently, an overlap analysis was performed using the R package VennDiagram for DE-lncRNAs and OS-related lncRNAs, whose common elements were labeled as OS-related DE-lncRNAs.

### Prediction of Target miRNAs and mRNAs of Oxidative Stress-Related Differentially Expressed-lncRNAs

Prediction of target miRNAs for OS-related DE-lncRNAs using Starbase and LncBase. Starbase (http://starbase.sysu.edu.cn/) is a friendly database for the prediction of target miRNAs by high-throughput CLIP-Seq experimental data and degradome experimental data. LncBase database (www.microrna.gr/LncBase) is a specialized database for recording lncRNA-miRNA interactions. The screening criteria of Starbase were as follows: low stringency ≥1; the screening criteria of LncBase was set to miTG-score ≥ 0.95; other parameters used the default parameters provided by the website. The final screening criteria for miRNAs were the miRNAs predicted by the combined Starbase and LncBase databases. we also used the miRWalk (http://mirwalk.umm.uni-heidelberg.de/) database to predict the target mRNAs of the predicted target miRNAs, which is a comprehensive database for predicting miRNA target genes (Dweep et al., 2011).

### Construction of the LncRNA-miRNA-mRNA Network

According to ceRNA regulation theory, lncRNAs are expressed in the same direction as mRNAs, while miRNAs and mRNAs & lncRNAs are expressed in the opposite direction. lncRNA-miRNA networks were identified using intersection analysis to identify common miRNAs between predicted targeting miRNAs and DE-miRNAs. In the same way, we obtained common mRNAs. After collating lncRNA-miRNA and miRNA-mRNA relationship pairs, the lncRNA-miRNA-mRNA networks were embellished using Cytoscape software, which is an open-source network visualization software platform mainly used for the analysis of complex biological networks. It can generate network structures and hierarchies for gene expression regulatory networks, protein interaction networks, and others.

### Gene Ontology and Kyoto Encyclopedia of Genes and Genomes Analysis of the mRNAs in the CeRNA Network

To predict the biological function of the ceRNA network of IDD, we performed GO analysis and KEGG pathway analysis on the mRNAs in the ceRNA network using ClusterProfiler (Yu et al., 2012). The screening conditions of GO analysis and KEGG pathway analysis were *p* < 0.05 and count ≥ 2.

### Protein-Protein Interaction Network Construction

The PPI information among all mRNAs in the ceRNA networks was identified using the search tool for the retrieval of interacting genes/proteins (STRING) online platform (http://www.string-db.org/) (Szklarczyk et al., 2019), and subsequently, their interactions were imported into the Cytoscape software to construct and visualize a PPI network. Then, the MCODE analysis was used to screen the top PPI networks.

### Identification of Key LncRNAs

We extracted all the lncRNA nodes and calculated their degrees in the ceRNA network. Candidate lncRNAs were selected based on degree >2. We also calculated the number of first relationship lncRNA-miRNA pairs and secondary relationship miRNA-mRNA pairs. The top five lncRNAs were candidate lncRNAs according to the descending order of the total number of first and second relationships. The common lncRNAs screened by both methods were identified as key lncRNAs. ceRNA sub-networks of each key lncRNA were extracted from the ceRNA network, and their relationships were demonstrated by the Sankey diagram plotted in the R package Ggalluvial. Moreover, in order to evaluate the diagnostic value of these four key lncRNAs, we constructed a nomogram and evaluated its diagnostic value using the ROC curve.

### Patient Preparation

Eleven patients (mean 55.1 age) with MRI-confirmed IDD and seven volunteers (mean 25.7 age, no clinical evidence of low back pain or sciatica) of fasting whole blood (collected from the left median cubital vein of each participant) samples were stored in the refrigerator at minus 80°C and collected to conduct the experiment (Table 1). Magnetic resonance imaging scans were performed to assess the extent of disc degeneration according to the Pfirrmann classification (Castro-Mateos et al., 2016). All IDD patients underwent surgery in the Second Affiliated Hospital of Shanxi Medical University, Taiyuan, Shanxi, China. The study was approved by the hospital Ethics Review Committee. In the study, informed consent was exempted. The ethical approval number of the clinical protocol is 2022YXNO.055.

**TABLE 1:** Basic information of the specimens.

Sample	Age	Gender	MRI grade
Control 1	25	M	II
Control 2	24	F	I
Control 3	29	M	I
Control 4	27	M	I
Control 5	30	F	II
Control 6	26	M	I
Control 7	28	M	I
IDD 1	53	F	IV
IDD 2	54	M	V
IDD 3	71	M	V
IDD 4	49	F	IV
IDD 5	80	F	V
IDD 6	28	M	IV
IDD 7	36	M	V
IDD 8	38	M	V
IDD 9	75	M	V
IDD 10	63	M	IV
IDD 11	59	F	V

M, male; F, male; MRI, magnetic resonance imaging.

### RNA Isolation and Quantitative Real-Time Polymerase Chain Reaction

A total of 18 whole blood samples were lysed with TRIzol Reagent (Life Technologies-Invitrogen, Carlsbad, CA, United States), and the total RNA was isolated following the manufacturer’s instructions. Then, the concentration and purity of the RNA solution were quantified using a NanoDrop 2000FC-3100 nucleic acid protein quantifier (Thermo Fisher Scientific, Waltham, MA, USALife Real). The extracted RNA was reverse-transcribed to cDNA using the SureScript-First-strand-cDNA-synthesis-kit (Genecopoeia, Guangzhou, China) prior to qRT-PCR. The qRT-PCR reaction consisted of 3 µl of reverse transcription product, 5 µl of 5 × BlazeTaq qPCR Mix (Genecopoeia, Guangzhou, China), and 1 µl each of forward and reverse primer. PCR was performed in a BIO-RAD CFX96 Touch TM PCR detection system (Bio-Rad Laboratories, Inc., United States) under the following conditions: initial denaturation at 95°C for 1 min, followed by 40 cycles that each involved incubation at 95°C for 20 s, 55°C for 20 s, and 72°C for 30 s. All primers (Table 2) were synthesized by Servicebio (Servicebio, Wuhan, China). The GAPDH gene served as an internal control, and the relative expression of four key lncRNAs was determined using the 2^−*ΔΔCt*^ method (Livak and Schmittgen, 2001). The experiment was repeated in triplicate on independent occasions. Statistical differences of four key lncRNAs between normal and IDD samples were detected by paired t-tests, using GraphPad Prism V6 (GraphPad Software, La Jolla, CA, United States), and the level of statistical significance was tested and represented as * for *p* < 0.05 and ** for *p* < 0.01.

**TABLE 2:** The primers for qRT-PCR.

Genes	Sequence (5′-3′)
MIR100HG	F: ATTTGGTGTATCGCTTCC
	R: CCCCTTTCTTTTCTCTT
LINC01359	F: TGA​AGA​GGT​AGC​AAG​AGA​GC
	R: GAG​GAT​GGA​AGG​ATA​GAT​GG
LUCAT1	F: GTGCTCGCTCTTGGTGA
	R: GGGGGGGAGTATGAAAC
GNAS-AS1	F: AGACCACAAAAGCATCCA
	R: GACCCAGCACAAAAACGG
GAPDH	F: CCC​ATC​ACC​ATC​TTC​CAG​G
	R: CAT​CAC​GCC​ACA​GTT​TCC​C

### Analysis of Immune Cell Characteristics

We further evaluated the infiltrating scores of 24 immune cells with the single-sample gene set enrichment analysis (ssGSEA) in the gsva R package (Rooney et al., 2015). Moreover, the relationships between immune cells and key lncRNAs were explored through Pearson correlation analysis.

### Statistical Analysis

All the statistics were done using the R software (version 4.0.2) and GraphPad Prism V6 (GraphPad Software, La Jolla, CA, United States). *p* < 0.05 was set as statistically significant for all the analyses.

## Results

### Identification of Intervertebral Disc Degeneration-Related Aberrantly Expressed Genes

**FIGURE 1:**
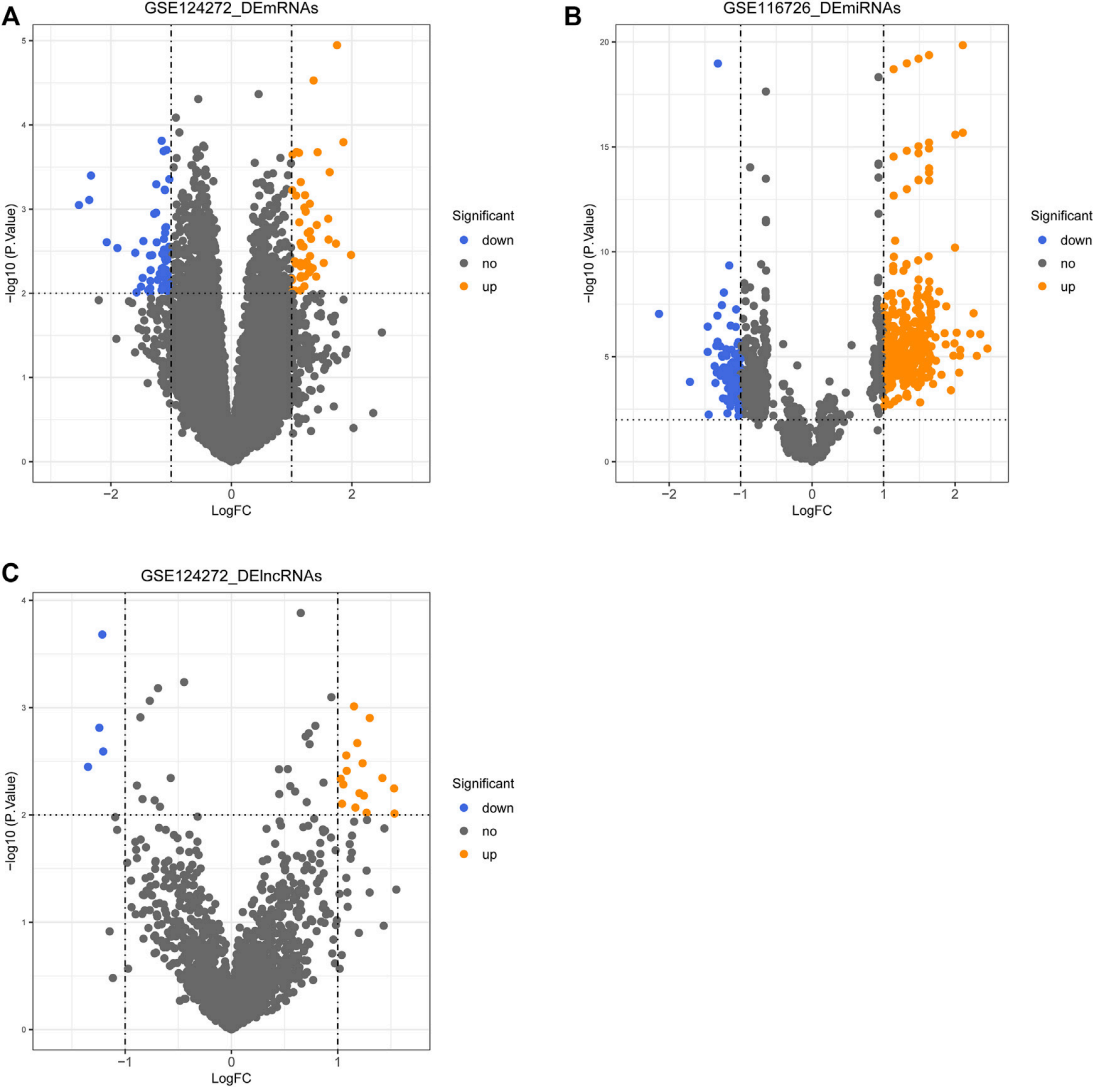
Volcano plot of differentially expressed genes (DEGs), lncRNAs (DE-lncRNAs), and miRNAs (DE-miRNAs) in the IDD group compared with the control group. **(A)** Volcano plot of DE-mRNAs. **(B)** Volcano plot of DE-lncRNAs. **(C)** Volcano plot of DE-miRNAs.

Differential expression analysis was performed using the R package limma based on transcriptomic data from the GSE124272 and GSE116726 datasets, with the significance threshold set at |log_2_ FC| ≥ 1 and *p* < 0.01. A total of 111 DE-mRNAs (Figure 1A, Supplementary Table S2) and 20 DE-lncRNAs were identified in the GSE124272 dataset (Figure 1B, Supplementary Table S3), with 52 mRNAs and 16 lncRNAs upregulated in the IDD group (*n* = 8) and 59 mRNAs and four lncRNAs downregulated in the IDD group (*n* = 8) compared with the control group (*n* = 8). miRNAs expression profiles were obtained from the GSE116726 dataset, containing three cases each of IDD and normal samples. A total of 502 DE-miRNAs were identified, of which, 402 miRNAs were expressed up-regulated in IDD samples and 100 miRNAs were expressed downregulated in IDD samples (Figure 1C, Supplementary Table S4).

### Analysis of Oxidative Stress-Related Differentially Expressed-LncRNAs-Mediated CeRNA Networks

**FIGURE 2:**
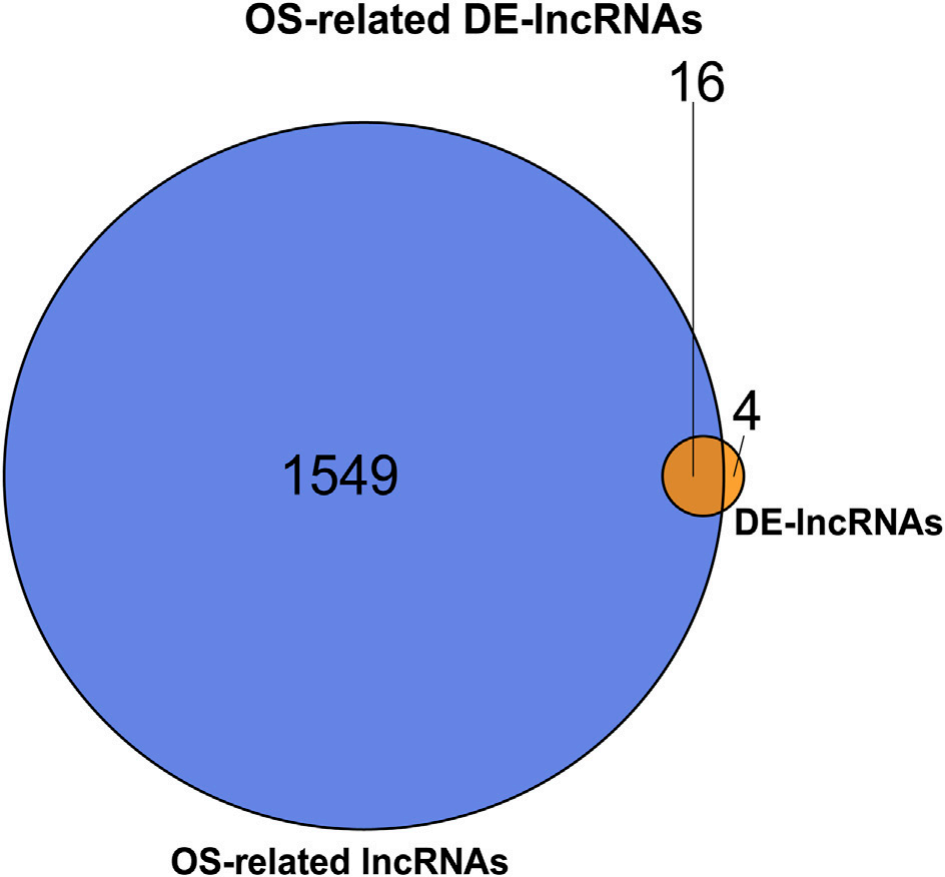
Venn diagram of common lncRNAs between OS-related lncRNAs and DE-lncRNAs.

After calculating the Spearman correlation of 2,129 OSGs with 43596 lncRNAs (from the GSE124272 dataset), a total of 1565 OS-related lncRNAs were identified based on a significance threshold of |cor| > 0.8 and *p* < 0.01 (Supplementary Table S5). Subsequently, an intersection analysis was performed using the R package VennDiagram to identify the common lncRNAs between OS-related lncRNAs and DE-lncRNAs. The results are shown in Figure 2, and a total of 16 OS-related DE-lncRNAs were identified, of which 14 were up-regulated and two were down-regulated in the IDD (Table 3).

**TABLE 3:** Differential expression of 16 OS-related DE-lncRNAs between normal and IDD groups.

Symbol	ID	logFC	*p*-value	Type
GRIK1-AS1	ENSG00000174680	1.533548116	0.009711464	Up
AADACL2-AS1	ENSG00000242908	1.529591952	0.005669244	Up
KCNJ2-AS1	ENSG00000267365	1.419842136	0.004527719	Up
NFE4	ENSG00000230257	1.271172161	0.009540417	Up
COL4A2-AS1	ENSG00000232814	1.245683747	0.006615357	Up
JMJD1C-AS1	ENSG00000272767	1.203987822	0.006267746	Up
MIR100HG	ENSG00000255248	1.183718415	0.002141863	Up
LINC00029	ENSG00000125514	1.165817974	0.008548733	Up
SYP-AS1	ENSG00000237341	1.152784528	0.000973094	Up
LINC01359	ENSG00000226891	1.084122661	0.003884443	Up
LUCAT1	ENSG00000248323	1.080241162	0.002788182	Up
MIR646HG	ENSG00000228340	1.052595815	0.005208917	Up
LINC00906	ENSG00000267339	1.040172614	0.007876342	Up
STARD7-AS1	ENSG00000204685	1.02798182	0.004603308	Up
RNF157-AS1	ENSG00000267128	−1.206265373	0.002561201	Down
GNAS-AS1	ENSG00000235590	−1.214634107	0.000208454	Down

**FIGURE 3:**
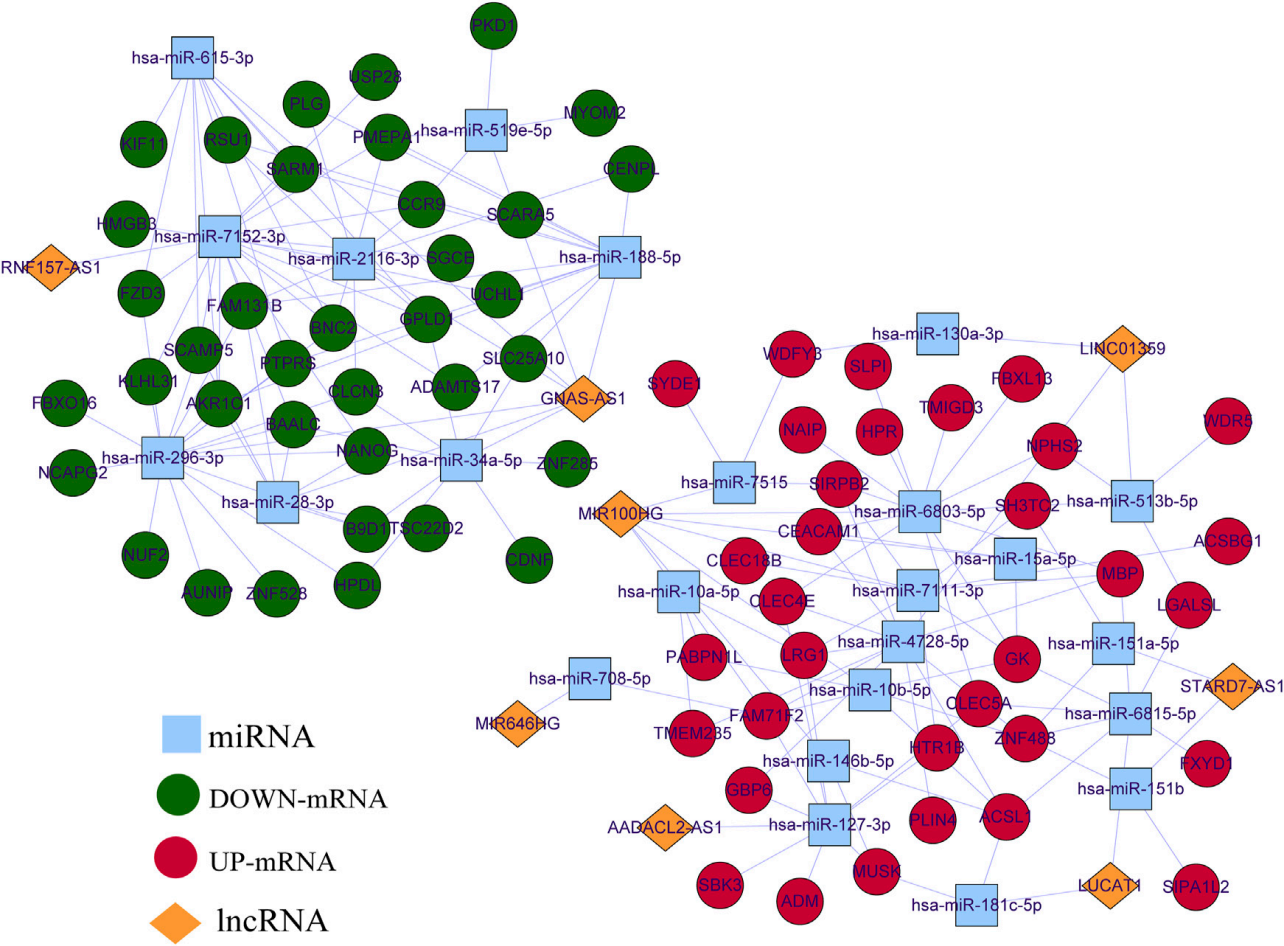
CeRNA network of OS-related DE-lncRNAs, DE-miRNA, and DE-mRNA. Red circle nodes represent up-regulated expression of mRNAs. Green circle nodes represent down-regulated expression of mRNAs. Yellow rhombus nodes represent upregulated expression of lncRNAs. Purple rhombus nodes represent downregulated expression of lncRNAs.Sky-blue square nodes represent DE-miRNAs.

Based on the prediction results from Starbase, LncBase, and miRWalk databases, after combining DE-miRNAs and DE-mRNAs according to ceRNA regulation theory, we finally visualized a ceRNA network containing eight OS-related DE-lncRNAs, 24 DE-miRNAs, and 70 DE-mRNAs by Cytoscape (Figure 3), which featured 183 edges (Supplementary Table S6) and a total of 159 ceRNA regulatory mechanisms (Supplementary Table S7). Specifically, lncRNA GNAS-AS1 (downregulated) could downregulate the expression of 35 mRNAs by competitive binding to seven DE-miRNAs (up-regulated); lncRNA RNF157-AS1 (downregulated) could simultaneously regulate the expression of 16 DE-mRNAs (downregulated) by competitive binding to hsa-miR-7152-3p (upregulated); lncRNA AADACL2-AS1 (upregulated) could act as a sponge for hsa-miR-127-3p (downregulated) to up-regulate the expression of nine mRNAs; lncRNA LINC01359 (upregulated) could regulate the expression of 16 DE-mRNAs (upregulated) by competitive binding with 3 DE-miRNAs (downregulated); lncRNA LUCAT1 (upregulated) regulated 8 DE-mRNAs (upregulated) through competitive binding to hsa-miR-181c-5p (downregulated) and hsa-miR-6815-5p (downregulated); lncRNA MIR100HG (upregulated) with 8 DE-miRNAs (downregulated) and 25 DE-mRNAs (upregulated) formed 38 ceRNA regulatory mechanisms; lncRNA STARD7-AS1 (upregulated) regulated the expression of three DE-mRNAs (upregulated) when acting as a sponge for hsa-miR-151a-5p (downregulated) and hsa-miR-151b (downregulated).

**FIGURE 4:**
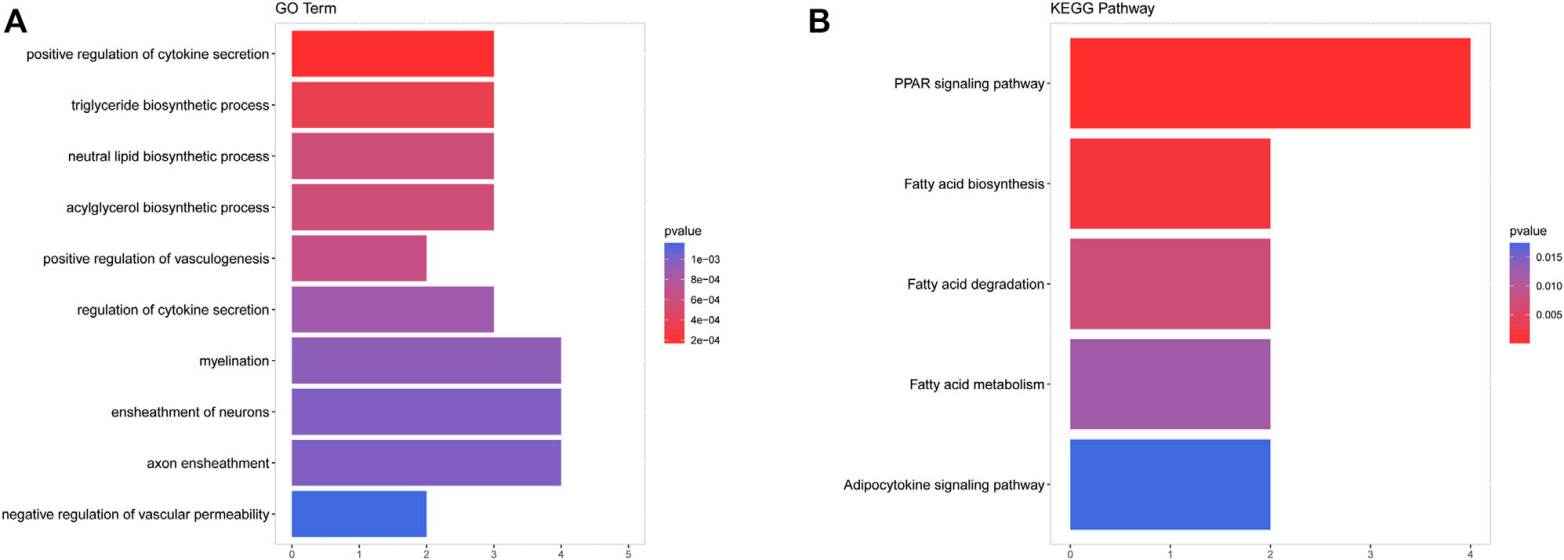
Functional enrichment of analysis of DE-mRNAs based on GO and KEGG. **(A)** The top 10 terms in the GO-BP category of GO analyses DE-mRNAs. **(B)** KEGG pathway analysis of DE-mRNAs.

**FIGURE 5:**
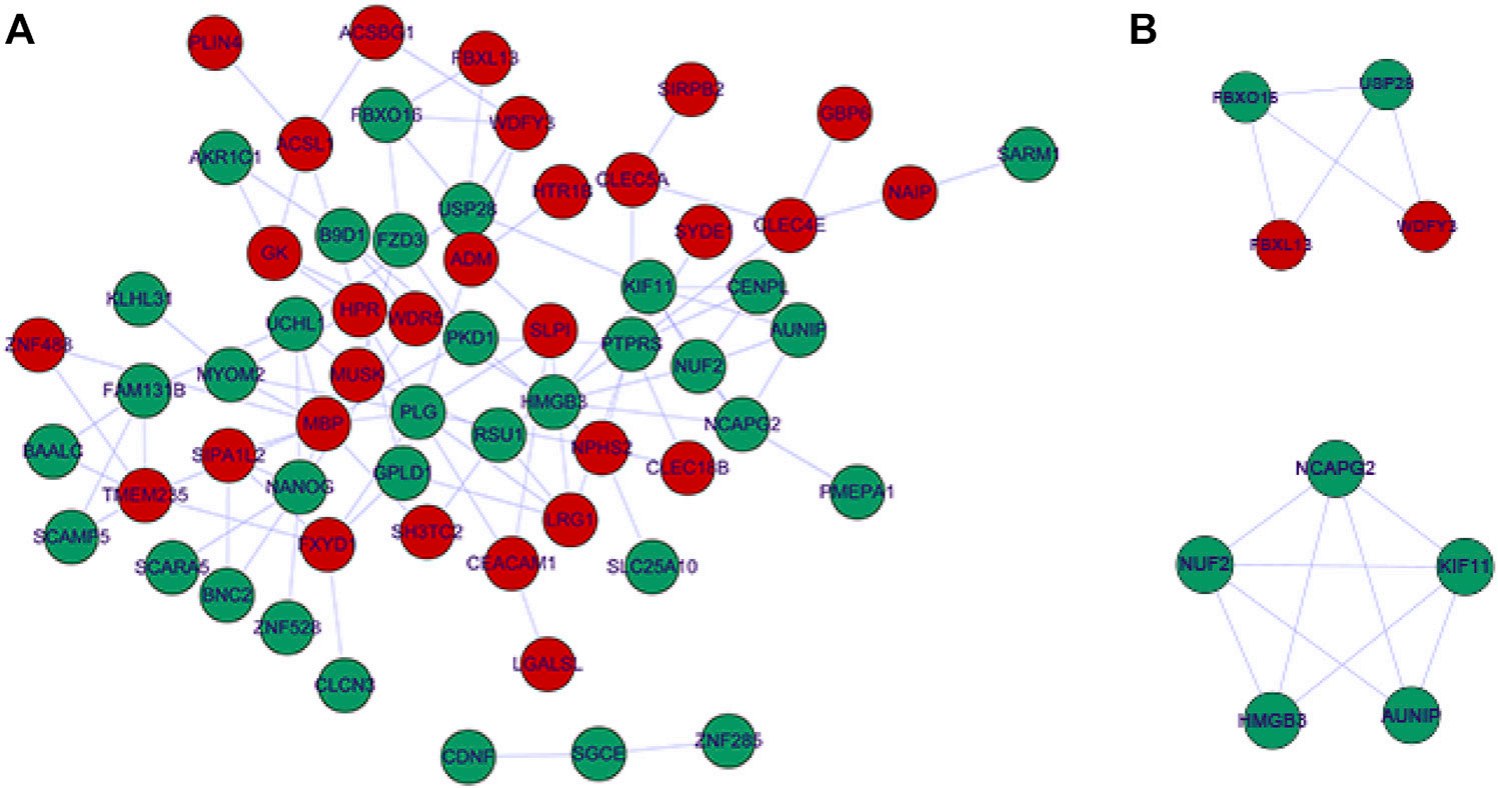
Protein-protein interaction network complex. **(A)** The protein-protein interaction network visualized by Cytoscape. Red nodes represent up-regulated expression, Green nodes represent up-regulated expression. **(B)** Top two PPI networks in MCODE analysis.

Subsequently, we performed functional enrichment analysis for 70 DE-mRNAs in the ceRNA network to initially explore the regulatory mechanisms of the eight OS-related DE-lncRNAs-mediated ceRNA networks. A total of 30 BP terms, 2 CC terms, and 11 MF terms were enriched by the GO system (Supplementary Table S8). In this study, which focused on BP categories, we found that these DE-mRNAs were closely related to the secretion of cytokines and their regulatory processes. Meanwhile, neuron-related terms such as ensheathment of neurons, axon ensheathment, and negative regulation of vascular permeability were significantly enriched, suggesting that ceRNA networks may be involved in regulating the pain or other proprioceptive sensations in IDD patients’ transmission. In addition, these DE-mRNAs were enriched in lipid-related biological processes, suggesting crosstalk between obesity and IDD. Moreover, angiogenic biological processes, which are considered to be the main cause of disc-related diseases, were significantly enriched. Figure 4A shows the top 10 terms in the GO-BP category. KEGG analysis revealed that these DE-mRNAs were significantly involved in the PPAR signaling pathway, fatty acid biosynthesis, degradation, and metabolic pathway, and adipocytokine signaling pathway (Figure 4B, Supplementary Table S9). Additionally, we explored the interaction relationships of 70 DE-mRNAs by the STRING tool. The confidence was set to 0.15 and after removing discrete proteins, finally, we could visualize a PPI network containing 63 nodes and 108 edges (Figure 5A). Moreover, we screened the top two clusters with the highest clustering scores (Figure 5B).

### Screening of Key Oxidative Stress-Related Differentially Expressed-LncRNAs

To select key lncRNAs from the ceRNA network, we performed the following analysis. The degree values of each lncRNAs in the ceRNA network were obtained by the NetworkAnalyst tool of Cytoscape software. The results are shown in Table 4, and we identified five nodes as candidate key lncRNAs (degree >2), including lncRNA MIR100HG, lncRNA GNAS-AS1, lncRNA LINC01359, lncRNA STARF7-AS1, and lncRNA LUCAT1 (all degree = 2). Meanwhile, we counted the number of first-relationship lncRNA-miRNA pairs and second-relationship miRNA-mRNA pairs. The results showed that lncRNA GNAS-AS1, MIR100HG, LINC01359, RNF157-AS1, and LUCAT1 were the top five lncRNAs (Table 5). Taken together, the four lncRNAs had higher degree values and more lncRNA-miRNA and miRNA-mRNA pairs, implying that these four lncRNAs were highly associated with the occurrence of IDD. The four lncRNAs were lncRNA GNAS-AS1 (GNAS Antisense RNA 1), lncRNA MIR100HG (Mir-100-Let-7a-2-Mir-125b-1 Cluster Host Gene), lncRNA LINC01359 (Long Intergenic Non-Protein Coding RNA 1359), lncRNA LUCAT1 (Lung Cancer Associated Transcript 1), which were defined as key lncRNAs.

**TABLE 4:** The degree values of each lncRNAs in the ceRNA network.

Rank	Gene name	Degree	Gene type
1	MIR100HG	7	lncRNA
2	GNAS-AS1	7	lncRNA
3	LINC01359	3	lncRNA
4	STARD7-AS1	2	lncRNA
5	LUCAT1	2	lncRNA
6	AADACL2-AS1	1	lncRNA
7	MIR646HG	1	lncRNA
8	RNF157-AS1	1	lncRNA

**TABLE 5:** The number of first-relationship lncRNA-miRNA pairs and second-relationship miRNA-mRNA pairs.

Rank	Gene name	lncRNA-miRNA pairs	miRNA-mRNA pairs	Total number
1	GNAS-AS1	7	67	74
2	MIR100HG	7	37	44
3	LINC01359	3	16	19
4	RNF157-AS1	1	16	17
5	LUCAT1	2	9	11
6	AADACL2-AS1	1	9	10
7	STARD7-AS1	2	4	6
8	MIR646HG	1	1	2

**FIGURE 6:**
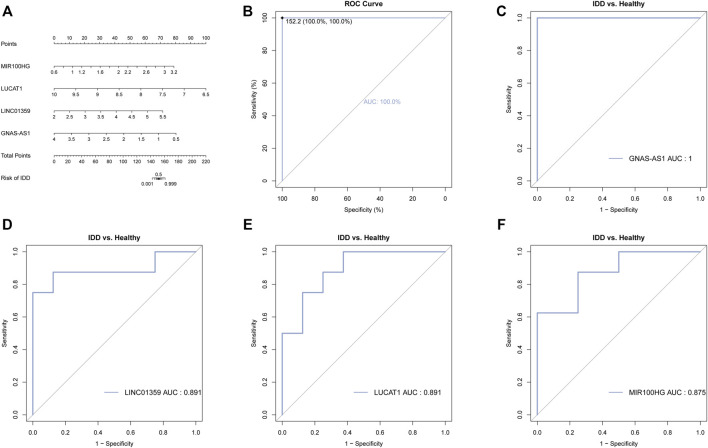
Diagnostic and prognostic value of the four key lncRNAs. **(A)** Nomogram of the four key lncRNAs. The value of each lncRNA was given a score on the point scale axis. A total score could be easily calculated by adding every single score and, by projecting the total score to the lower total point scale, we were able to estimate the probability of IDD. **(B)** The calibration curve for the nomogram. **(C)** ROC analysis of GNAS-AS1. **(D)** ROC analysis of LINC01359. **(E)** ROC analysis of LUCAT1. **(F)** ROC analysis of MIR100HG.

In order to evaluate the diagnostic value of these four key lncRNAs, we constructed a nomogram and evaluated its diagnostic value using the ROC curve. We found that the AUC value of these four key lncRNAs and nomogram was greater than 0.8, indicating that these four key lncRNAs had high diagnostic value (Figure 6).

### Construction of LncRNA-miRNA-mRNA Subnetworks

**FIGURE 7:**
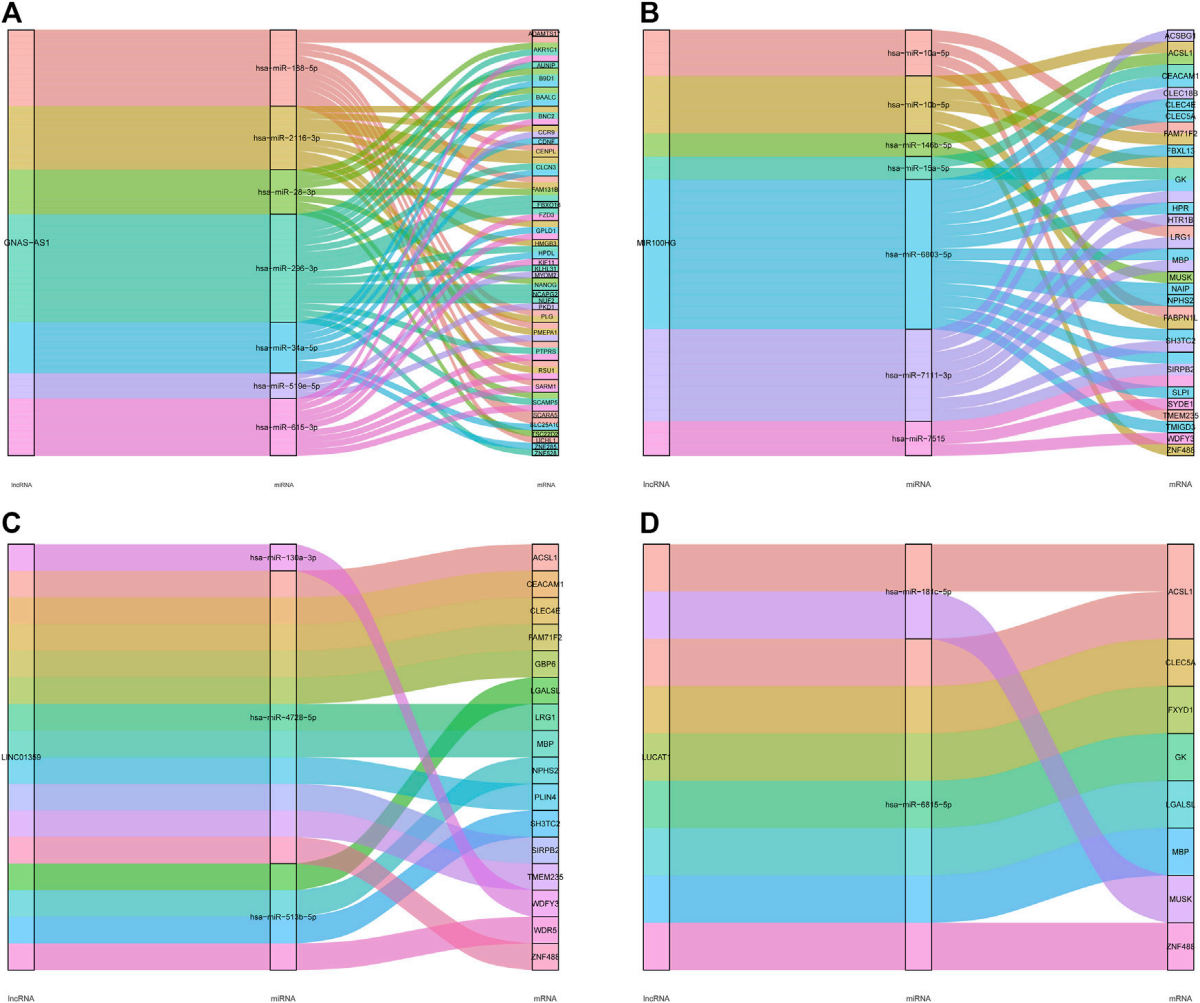
LncRNA-miRNA-mRNA subnetwork of the four key lncRNAs. **(A)** The lncRNA GNAS-AS1-miRNA-mRNA network. **(B)** The lncRNA MIR 100HG-miRNA-mRNA network. **(C)** The lncRNA LINC01359-miRNA-mRNA network. **(D)** The lncRNA LUCAT1-miRNA-mRNA network.

We selected four key lncRNAs related to IDD, extracted the miRNAs and mRNAs related to them, and reconstructed the corresponding lncRNA-miRNA-mRNA subnetwork. The lncRNA GNAS-AS1-miRNA-mRNA network was composed of one lncRNA node, seven miRNA nodes, 35 mRNA nodes, and 67 edges (Figure 7A). The lncRNA MIR100HG-miRNA-mRNA subnetwork consisted of one lncRNA node, seven miRNA nodes, 25 mRNAs, and 37 edges (Figure 7B). The lncRNA LINC01359-miRNA-mRNA subnetwork consisted of one lncRNA node, three miRNA nodes, 16 mRNA nodes, and 16 edges (Figure 7C). The lncRNA LUCAT1-miRNA-mRNA subnetwork consisted of one lncRNA node, two miRNA nodes, eight mRNA nodes, and nine edges (Figure 7D).

### Verification of Four Key LncRNAs

**FIGURE 8:**
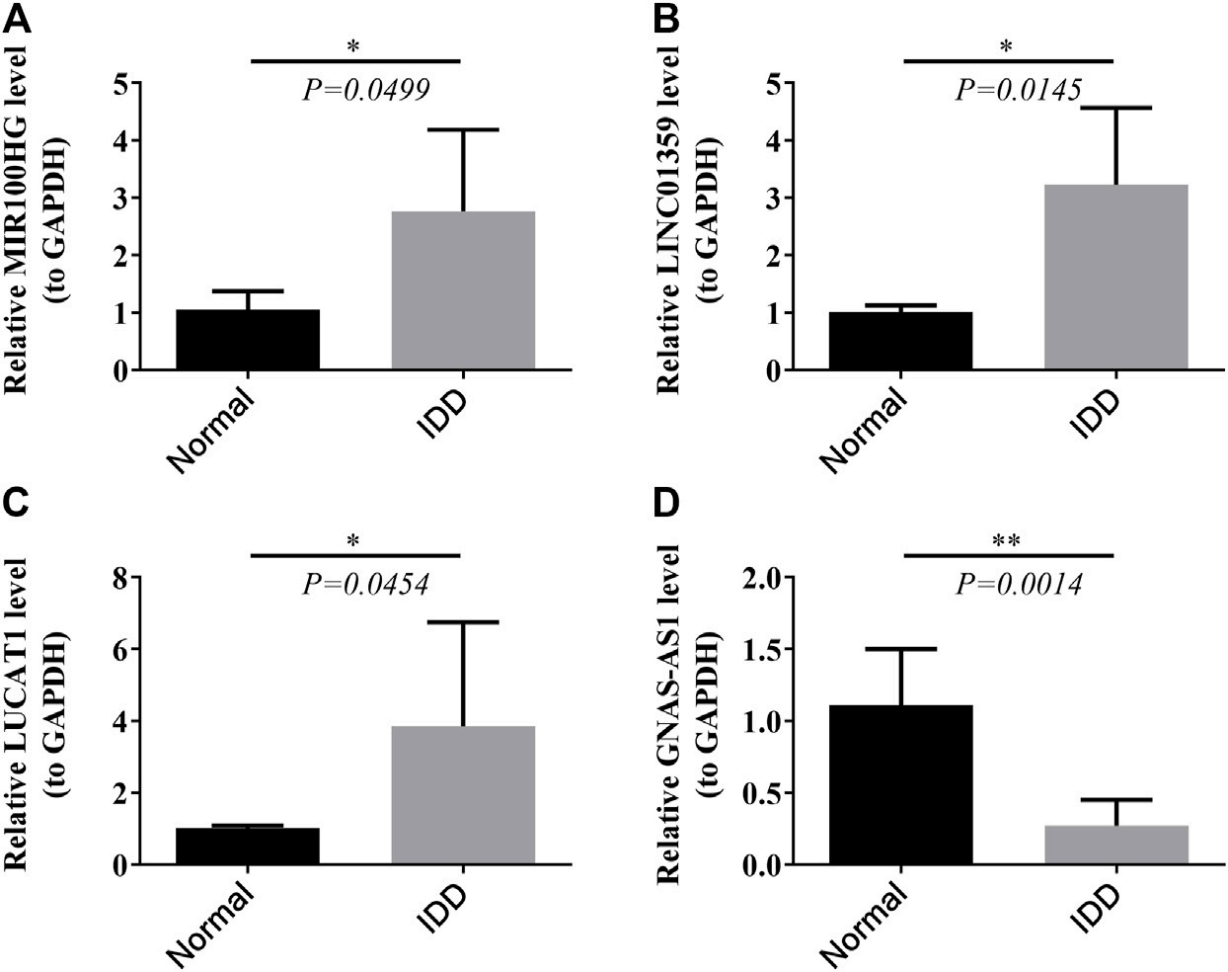
Relative expression of four key lncRNAs MIR 100HG **(A)**, LINC01359 **(B)**, LUCAT1 **(C)**, and GNAS-AS1 **(D)** in clinical samples by RT-PCR.

To verify the authenticity of the key lncRNAs we identified, we selected eleven blood samples from IDD patients and seven blood samples from healthy control subjects for qRT-PCR molecular validation. The results have shown significant differences (*p* < 0.05) in the relative expression of all key lncRNAs between IDD patients and healthy control subjects, as shown in Figure 8. Compared with healthy subjects, lncRNAs MIR100HG, LINC01359, and LUCAT1 were significantly upregulated in the IDD group (Figures 8A–C), whereas the expression level of lncRNA GNAS-AS1 was significantly downregulated in the IDD group (Figure 7D), and these results were consistent with bioinformatic analysis (Table 3).

### Association of Key LncRNAs With Immune Infiltrating Cells

**FIGURE 9:**
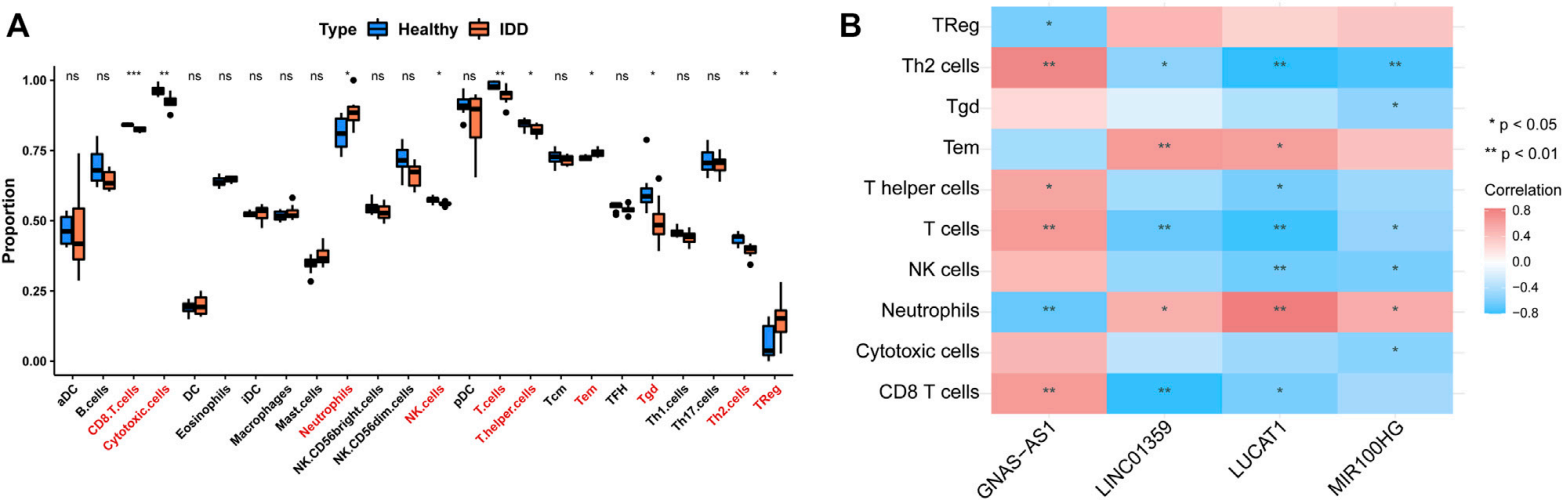
Single sample GSEA and Pearson correlation analysis of immune infiltrating cells. **(A)** The proportion of immune infiltrating cells in IDD and healthy group. **(B)** The correlation of the above 10 differentially abundant immune infiltrating cells with four key lncRNAs.

**FIGURE 10:**
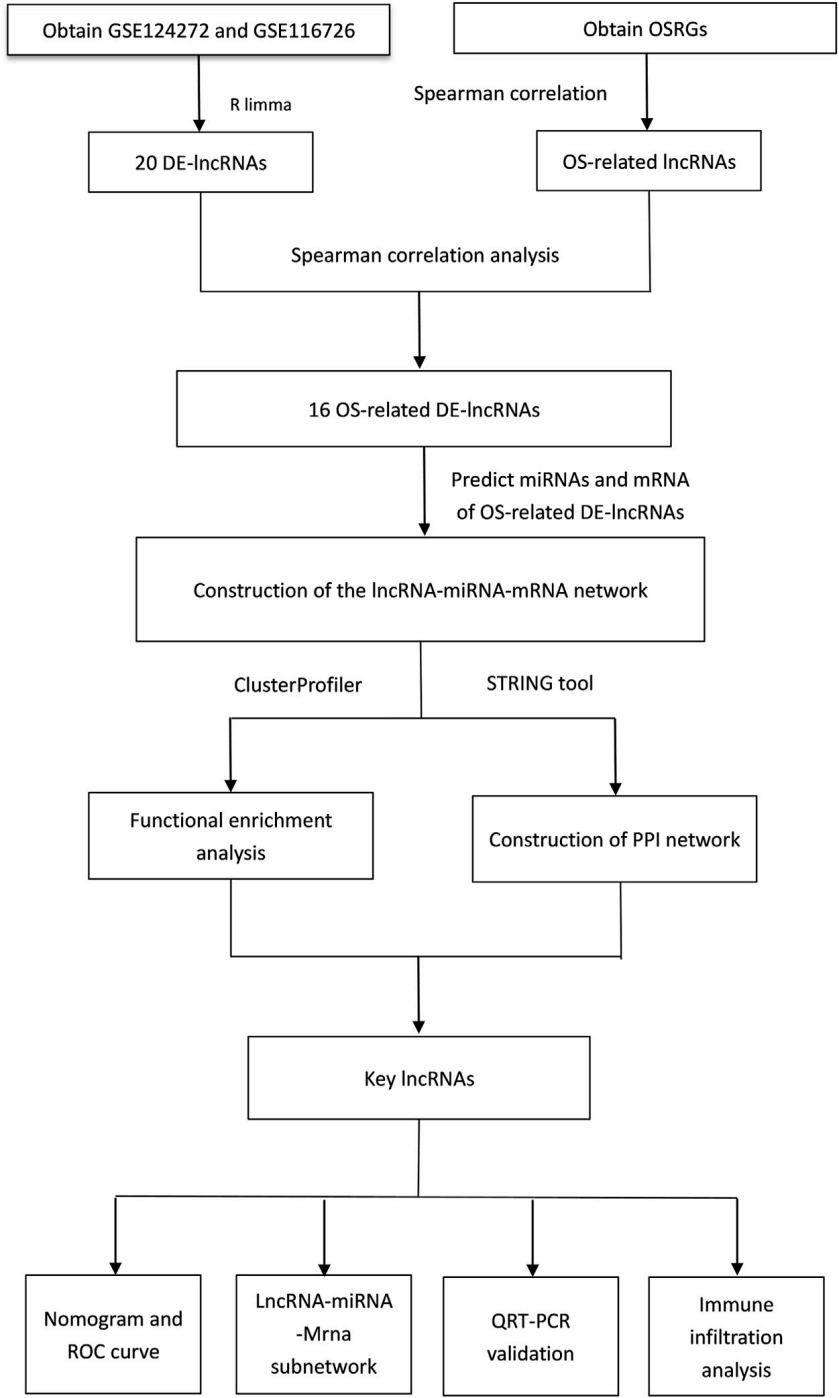
Flowchart of key lncRNAs associated with oxidative stress were identified by GEO database data and whole blood analysis of intervertebral disc degeneration patients.

We assessed the abundance of immune infiltrating cells in IDD (*n* = 8) and normal (*n* = 8) samples from the GSE124272 dataset using ssGSEA. By Wilcoxon rank-sum test, we found that the abundance of neutrophils, Tem, and TReg was significantly higher in the IDD group than in the normal group; whereas CD8 T cells, cytotoxic cells, NK cells, T cells, T helper cells, Tgd, and Th2 cells in the normal group were accounted for a higher percentage (Figure 9A). Subsequently, we calculated the Pearson correlation of four key lncRNAs with the above 10 differentially abundant immune infiltrating cells. The results were shown in Figure 9B. Specifically, lncRNA GNA-AS1 was moderately negatively correlated with Neutrophils (cor = −0.68) and TReg (cor = −0.61), while moderately or strongly positively correlated with T helper cells (cor = 0.59), T cells (cor = 0.65), CD8 T cells (cor = 0.65), and Th2 cells (cor = 0.78); lncRNA LINC01359 was moderately or strongly negatively correlated with Th2 cells (cor = −0.54), T cells (cor = −0.67), and CD8 T cells (cor = −0.78), and with Neutrophils (cor = 0.52) and Tem (cor = 0.66) showed moderate positive relationship; lncRNA LUCAT1 was associated with Th2 cells (cor = −0.80), T cells (cor = −0.76), NK cells (cor = −0.63), T helper cells (cor = −0.61), and CD8 T cells (cor = −0.61) showed moderate or strong negative correlation and moderate or strong positive correlation with Neutrophils (cor = 0.84) and Tem (cor = 0.62); lncRNA MIR100HG correlated with Th2 cells (cor = −0.74), NK cells (cor = −0.60), and Cytotoxic cells (cor = −0.55), Tgd (cor = −0.53), and T cells (cor = −0.52) with moderate or strong negative correlations, and with Neutrophils (cor = 0.54) with moderate positive correlations. The research process of this study is as follows (Figure 10).

## Discussion

As we know, a serious imbalance between endogenous and exogenous reactive oxygen species and the body’s antioxidant defense system can lead to oxidative stress (OS).OS plays an important role in disc degeneration, causing disc cell death and extracellular matrix degeneration (Zhang et al., 2020). An increasing number of studies have demonstrated that some non-coding RNAs, such as circRNAs, LncRNAs and micro RNAs (miRNAs), serve as ceRNAsthat regulate IDD initiation and progression (Li et al., 2018; Zhan et al., 2019). Analysis of the characteristics of OSRG associated with IDD, as well as the regulatory mechanisms of ceRNA and immune infiltration analysis, may help researchers to distinguish IDD and reveal the underlying mechanisms (Gámez-Valero et al., 2020).

In this study, we conducted functional enrichment analysis of 70 DE-mRNAs in the ceRNA network, and preliminarily explored the regulatory mechanism of eight OS-related DE-lncRNAS mediated ceRNA networks. GO enrichment analyses were conducted to find these DE-mRNAs were closely related to the secretion of cytokines and their regulatory processes. In IDD, cytokines production is increased. Production of cytokines is more common in degenerative discs than in normal discs (Roberts et al., 2006). There is also evidence that cytokines may be the cell nucleus pulposus-induced nerve lesions of factors, such as inflammatory cytokines IL-1β and TNF-α, are considered to be the key mediators of IDD that can effectively accelerate the IDD progress, and they also upregulated in the IDD. They are closely related to the various pathological processes of IDD, including inflammation, matrix damage, cell aging, autophagy, and apoptosis (Battié et al., 2009; Yang et al., 2020). Meanwhile, neuron-related terms such as ensheathment of neurons, axon ensheathment, and negative regulation of vascular permeability were significantly enriched. Some studies suggest that the growth of sensory afferent fibers into the disc may lead to discgenic pain after IDD (Leimer et al., 2019; Zhang et al., 2021). Fiber wrapping, changes in axon distance, and loss of myelin sheath can lead to neural structural abnormalities that may lead to the onset of pain and the exacerbation of chronic pain (Brifault et al., 2020). In addition, these DE-mRNAs were enriched in lipid-related biological processes, suggesting crosstalk between obesity and IDD. In addition to mechanical effects, obesity has important metabolic and inflammatory effects on the homeostasis of the intervertebral disc environment, which are mediated by adipokines (Ruiz-Fernández et al., 2019). Leptin, the most common adipokines, and adiponectin have a significant effect on the internal environment of the intervertebral disc. Adipose tissue can cause a systemic inflammatory state through secretion of pro-inflammatory adipocytokine, leptin (Sharma, 2018). Leptin can regulate proteomes through different signal transduction pathways. In contrast, adiponectin has been reported to play an anti-inflammatory role in diseased disc tissue (Sharma, 2018; Brifault et al., 2020). Moreover, angiogenic biological processes, which are considered to be the main cause of disc-related diseases. Disc degeneration is associated with angiogenesis. Degenerative disc disease is thought to be characterized by angiogenesis and increased expression of vascular endothelial growth factor (VEGF). Angiogenesis affects the pain intensity of disc herniation and negatively affects postoperative pain improvement, mobility, and overall quality of life (Lee et al., 2011; Sheng et al., 2018). KEGG analysis revealed that these DE-mRNAs were significantly involved in the PPAR signaling pathway, fatty acid biosynthesis, degradation, and metabolic pathway, and adipocytokine signaling pathway. These pathways are associated with obesity (Mao et al., 2021).

In this study, we identified four differentially expressed lncRNAs associated with oxidative stress. These are lncRNA GNAS-AS1, lncRNA MIR100HG, lncRNA LINC01359, lncRNA LUCAT1. GNAS-AS1 has been reported to promote tumor progression in non-small cell lung cancer (NSCLC) by altering macrophage polarization via the GNAS-AS1/mir-4319/NECAB3 axis (Li et al., 2020b). GNAS-AS1 plays a carcinogenic role by mediating β-catenin expression and may be an important gene involved in the formation and progression of nasopharyngeal carcinoma (Wang et al., 2020). Overexpression of Mir-185 Inhibits disc degeneration by inactivating the Wnt/β -catenin signaling pathway and galactose agglutinase-3 (Yun et al., 2020). Although there are no studies on the relationship between GNAS-AS1 and Wnt/β -catenin signaling Pathway, we have reason to believe that GNAS-AS1 can affect the process and development of IDD by regulating Wnt/β -catenin Signaling Pathway. GNAS-AS1 has also been reported as a prognostic indicator of osteosarcoma (Mi et al., 2021). MIR100HG promotes the development of triple-negative breast cancer (TNBC) by regulating the P27 gene and sponging mir-5590-3p. MIR100HG regulates p27 gene to control cell cycle. In G1 phase, knockdown of MIR100HG reduced cell proliferation and induced cell stagnation. Overexpression of MIR100HG significantly increased cell proliferation, and mir-5590-3p expressed the opposite effect (Wang et al., 2018b; Chen et al., 2020). The lack of the P27 gene activates the expression of Shh signaling pathway and promotes the proliferation of osteoblasts, thus playing a role in promoting IDD (Liu et al., 2017). MIR100HG may promote the proliferation, migration, and invasion of laryngeal squamous cell carcinoma (LSCC) through down-regulation of mir-204-5p (Huang et al., 2019). It is also associated with ductal adenocarcinoma of the pancreas and colorectal cancer (Ottaviani and Castellano, 2018; Peng et al., 2022). LINC01359 may be linked to hepatocellular carcinoma (HCC), that’s all we got (Tan et al., 2019). LUCAT1 is not available in PubMed, but the silencing of hsa-miR-181c-5p can inhibit the proliferation and promote the apoptosis of nucleus pulposus cells (Meng and Xu, 2021). We revealed a direct association between these four key lncRNAs and IDD, it may be important for the pathogenesis and clinical treatment exploration of IDD, which will be our focus in the future.

In addition to the above regulatory mechanisms, patterns of immune infiltration are also very important. Neutrophils, a type of white blood cell, are the most abundant in white blood cells, accounting for about 50%–70%, and are an important part of innate immunity. It has been suggested that neutrophils accumulate in IDD tissues and directly act on degenerative intervertebral discs by releasing relevant inflammatory factors, forming a vicious cycle. Neutrophils were significantly associated with IDD progression (Li et al., 2022). This was also consistent with our results that neutrophils were significantly higher in the IDD group than in the normal group. Effector memory T cell (Tem) is a type of T cell that performs immune protection by rapidly producing effector cytokines. NK cells and Tem secrete IL-4 and IFN-γ to play an immune role. Regulatory T cells (Tregs) are a subgroup of immunosuppressive T cells that secrete anti-inflammatory cytokines such as IL-4 and IL-10 to suppress inflammatory responses. It has been shown that both IFN-γ and IL-4 are elevated in degenerative intervertebral discs, and IFN-γ affects IDD by inducing the release of inflammatory cytokines and increasing ICAM-1 expression (Gabr et al., 2011; Risbud and Shapiro, 2014). T cell is the main component of lymphocytes and is a very important group in human immunity. There are a lot of different types of T cells, based on different criteria. Helper T cells are the largest subgroup of T cells, with many types and different functions. Th2 is one of the helper T cells, and the effector molecules are IL-4, IL-5, IL-10, and IL-13. Studies have shown that IL-10 reduction can accelerate disc degeneration in animal models (Wang et al., 2018a). A series of studies have shown that intervertebral disc degeneration can be alleviated by inhibiting IL-13, as well as blocking the associated cellular signaling pathways and inhibiting fibrosis in tissues (Van Dyken and Locksley, 2013; Li et al., 2019). This is different from our experimental results, which may be related to the number of different effect factors. CD8 was expressed in 30%–50% T cells and differentiated into cytotoxic T cells (CTL) after activation. Usually called CD8+T cells are CTLs, its main function is to kill target cells. Recent studies have indicated that CD8 T cells in rat IDD models are more prone to apoptosis, and CD8 T cells play a role in the pathogenesis of IDD and apoptosis (Li et al., 2020a; Cao et al., 2021). T cells gamma delta (Tgd) is a unique group of lymphocytes that are often enriched on the surface of epithelial cells. It regulates not only inflammation, but also autoimmune diseases (Yeung et al., 2000; Paul et al., 2014). It may play a role in IDD by modulating immune and inflammatory responses (Ma et al., 2022).

There are some limitations to this experiment. The sample size of qRT-PCR is insufficient, so the sample size should be increased for further study. None of the four key lncRNAs we obtained has been reported in IDD.

## Conclusion

In this study, DE-lncRNAs, DE-miRNAs, DE-mRNAs, and OS-related DE-lncRNAs in the IDD group and control group were obtained through the GEO database and GeneCards database. The new four key lncRNAs were screened by constructing a ceRNA network, namely lncRNA GNAS-AS1, lncRNA MIR100HG, lncRNA LINC01359, and lncRNA LUCAT1, and further validated by qRT-PCR. We also identified 10 immune infiltrating cells associated with key lncRNAs, suggesting that extensive infiltration of neutrophils, Tem, and Tregs may be associated with the development of IDD. The four new lncRNAs identified by us can provide help for the early diagnosis and treatment of IDD. We will continue to pay attention to the role of these lncRNAs.

## Data Availability

The original contributions presented in the study are included in the article/Supplementary Material, further inquiries can be directed to the corresponding author.
